# Suspected Japanese Pieris (*Pieris japonica*) Poisoning in an Alpaca (*Vicugna pacos*)

**DOI:** 10.3390/vetsci12090806

**Published:** 2025-08-25

**Authors:** Saki Tanaka, Haruka Takimoto, Yuki Matsubara, Tsunenori Tsujimoto, Jun Sasaki

**Affiliations:** 1 School of Veterinary Medicine, Iwate University, 3-18-8 Ueda, Morioka 020-8550, Japan; 2 Morioka Zoological Park ZOOMO, Morioka 020-0803, Japan

**Keywords:** *Pieris japonica*, Japanese pieris poisoning, alpaca, zoo animal, toxicology

## Abstract

A zoo-housed alpaca was found recumbent with profuse frothy salivation and an inability to stand. Supportive treatment, including intravenous fluid therapy via the jugular vein and oral administration of activated charcoal, was initiated. Despite these interventions, the animal’s condition progressively worsened, with clinical signs including vomiting and neurological manifestations such as paddling movements and opisthotonus. The alpaca died approximately 4 h after discovery by first observation of clinical signs. Necropsy revealed a large volume of white, foamy fluid present in the tracheal and bronchial lumens. The lungs were dark red and markedly congested and edematous throughout all lobes. Multiple ecchymotic hemorrhages were observed on the mucosal surface of the pyloric region of the third compartment of the stomach and on the serosal surface of the duodenum. Histopathological examination revealed severe pulmonary congestion and edema, along with marked congestion of the liver, spleen, and kidneys. The gastric contents were green and contained plant material, including ten leaves morphologically identified as Japanese pieris (*Pieris japonica*). Postmortem inspection of the enclosure revealed the presence of Japanese pieris shrubs with evidence of browsing. Based on these findings, acute poisoning from ingestion of Japanese pieris was diagnosed.

## Simple Summary

An alpaca housed in a zoo was suddenly found unable to stand, salivating heavily, and showing signs of distress. Despite receiving emergency medical care, including fluids and activated charcoal, the animal’s condition rapidly worsened and it died within a few hours. A detailed examination after death showed serious damage to the lungs and other organs. Plant material found in the stomach included leaves identified as Japanese pieris (*Pieris japonica*), a known toxic plant. Shrubs of this plant were also found inside the alpaca’s enclosure. This case highlights the risk of accidental poisoning from ornamental plants and the importance of careful environmental management in zoo settings.

## 1. Introduction

Japanese pieris (*Pieris japonica*), an evergreen broadleaf shrub native to Japan, belongs to the family Ericaceae and is widely distributed throughout most regions of Japan, excluding Hokkaido. All parts of the plant—including leaves, flowers, stems, and seeds—contain toxic compounds, primarily grayanotoxins (GTXs) [1]. Cases of Japanese pieris poisoning have been documented in various animal species, including goats and sheep [2,3,4,5,6]. In this report, we described a case of spontaneous Japanese pieris poisoning in a zoo-housed alpaca (*Vicugna pacos*), with particular focus on the pathological findings.

## 2. Case Presentation

The case involved a 10-year-old, 70.4 kg female alpaca. Since May 2020, the animal had been undergoing treatment for a *Cheyletiella* mite infestation on the medial aspects of both fore- and hindlimbs, but otherwise had no significant medical history. On 5 April 2023, the alpaca was introduced for the first time into a newly constructed outdoor enclosure. Later that day, it was found recumbent with profuse frothy salivation. Blood samples collected after the onset of clinical signs revealed findings summarized in Table 1 [7]. Laboratory results showed marked hyperglycemia and severe hypophosphatemia, along with mild hypocalcemia, mild hypokalemia, and a slight elevation in creatine kinase levels. Emergency supportive care was initiated, including gastric intubation, intravenous fluid therapy (Lactated Ringer’s solution with glucose and 0.9% sodium chloride solution), and oral administration of activated charcoal (1.2 g, Nefguard Karyu, Kyoritsu Seiyaku, Tokyo, Japan). Gastric lavage was attempted via a gastric tube; however, only 10 mL of gastric fluid was recovered, indicating limited effectiveness. Despite these interventions, the animal’s neurological condition progressively deteriorated approximately 2 h after onset, manifesting vomiting, limb paddling, opisthotonus, and Cheyne–Stokes respiration. Although resuscitation efforts, including oxygen supplementation, administration of diazepam, aminophylline hydrate, dimorpholamine, hydrocortisone sodium succinate, and adrenaline, as well as chest compressions, the alpaca died approximately 4 h after being observed with clinical signs. A necropsy was performed the following day at the zoo facility, and major organs collected during the procedure were submitted to our laboratory for further examination.

**Figure 1:**
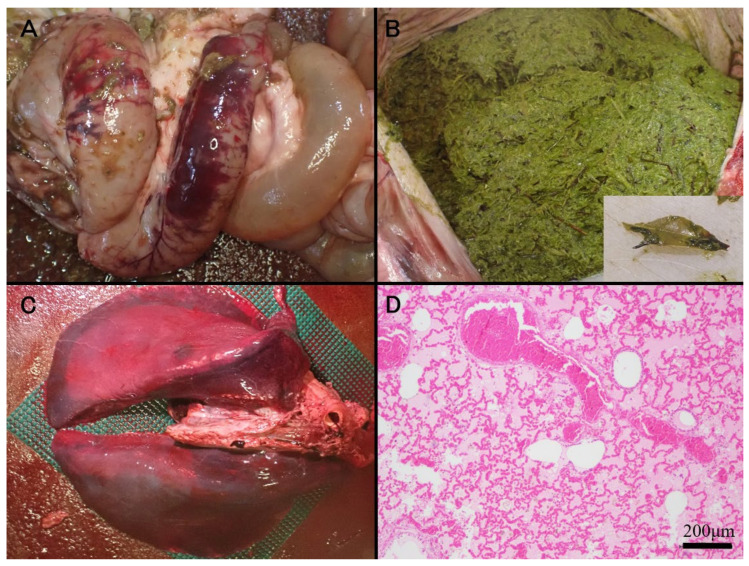
(**A**) Ecchymotic hemorrhages on the serosal surface of the duodenum. (**B**) The gastric lumen filled with green ingesta containing ten leaves identifiable as Japanese pieris foliage (inset). (**C**) Severe congestion in all lung lobes. (**D**) Prominent congestive edema in the lung (HE).

Gross examination revealed ecchymotic hemorrhages on the mucosal surface of the pyloric region of the third stomach compartment and on the serosal surface of the duodenum (Figure 1A). The gastric lumen was filled with green material, including ten leaves morphologically consistent with Japanese pieris (Figure 1B). The leaves are morphologically characterized by an oblong-to-lanceolate shape, measuring 5–8 cm in length, with a thick, green lamina and a glossy adaxial surface. The lungs appeared diffusely dark red across all lobes (Figure 1C), exhibiting marked congestion and edema. A white, foamy fluid was present within the lumens of the trachea and bronchi. Additionally, the liver, spleen, and kidneys showed generalized reddish discoloration consistent with congestion, and dilation of the right atrium and ventricle was also noted.

**Figure 2:**
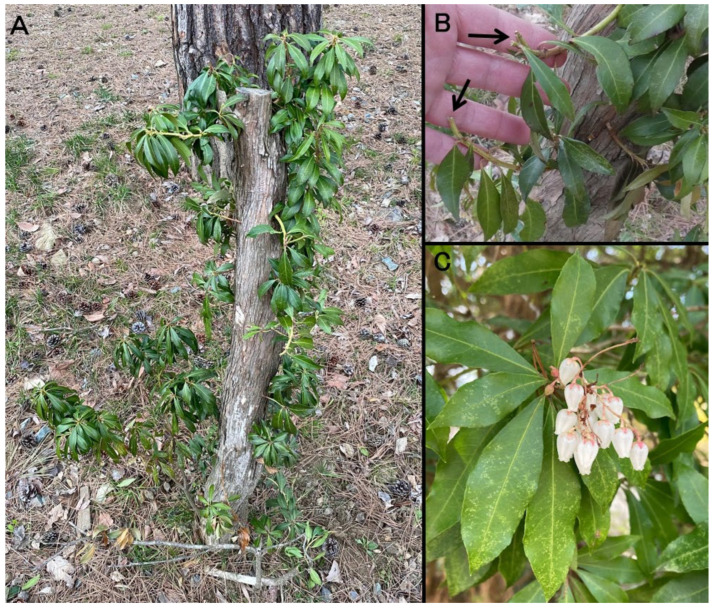
(**A**) Japanese pieris identified within the paddock. (**B**) Evidence of browsing (arrows) observed at multiple locations on the Japanese pieris. (**C**) Japanese pieris flowers within the same paddock.

Tissue samples collected during necropsy were fixed in 10% neutral-buffered formalin, routinely processed, embedded in paraffin, and sectioned at 4 μm using a microtome. The sections were stained with hematoxylin-eosin (HE) and examined histologically. Histopathological examination of the lungs revealed extensive and severe alveolar edema, accompanied by marked congestion in numerous blood vessels, including capillaries within the alveolar walls (Figure 1D). In the spleen, the red pulp exhibited pronounced congestion and evidence of extramedullary hematopoiesis. The liver showed severe congestion within the sinusoids and larger vessels, with scattered brownish pigments presumed to represent hemosiderin in the cytoplasm of hepatocytes. Marked congestion was also observed in both cortical and medullary vessels of the kidneys. In contrast, histological evaluation of the gastrointestinal mucosa and cerebrum was limited owing to advanced postmortem autolysis. Following the animal’s death, inspection of the outdoor enclosure revealed Japanese pieris shrubs with signs of browsing (Figure 2). The total quantity of ingested plant material could not be determined, and chemical analysis of the gastric contents for specific toxins was not performed.

## 3. Discussion

The principal pathological findings in this case included severe pulmonary congestion and edema, accompanied by ecchymotic hemorrhages on the mucosal surface of the pyloric region of the third stomach compartment and the serosal surface of the duodenum. Additionally, marked congestion was observed in the liver, spleen, and kidneys, indicating systemic circulatory disturbances involving multiple organs. These pathological features are consistent with those previously reported in cases of Japanese pieris poisoning in goats and sheep [2,3,4,5,6]. The acute clinical course, culminating in sudden death and lacking lesions suggestive of alternative causes, further supports the conclusion that mortality resulted from acute intoxication with toxic compounds contained in Japanese pieris. The subsequent discovery of Japanese pieris shrubs showing clear evidence of browsing within the enclosure after the animal’s death provides additional support for a causal relationship with this toxic plant. Although aspiration pneumonia has been documented as a primary cause of death in certain Japanese pieris poisoning cases in goats [5], no histopathological evidence of aspiration pneumonia was observed in the present case.

Japanese pieris, a member of the Ericaceae family, is an evergreen broadleaf shrub native to Japan and is widely distributed throughout the country, with the exception of Hokkaido. The entire plant, including leaves and seeds, contains various toxic compounds, among which GTXs—a group of diterpenoid compounds—are the most notable [1]. GTXs exert their toxic effects by binding to voltage-gated sodium (Na^+^) channels on cell membranes, resulting in persistent channel activation and prolonged membrane depolarization. This mechanism leads to sustained excitation of nerve and muscle cells [8]. Several structural isomers of GTXs have been identified, each displaying distinct degrees of toxicity and pharmacological activity, with GTX I, GTX II, and GTX III being the most extensively characterized [9]. Because Japanese pieris is native to Japan, research on its toxic constituents has predominantly been conducted domestically [1,9,10,11,12]. A related human intoxication syndrome, known as “mad honey” poisoning, results from ingestion of honey derived from the nectar of toxic Ericaceae species and is well documented along the eastern Black Sea coast, particularly in Turkey. This syndrome is characterized by pronounced neurotoxic and cardiotoxic effects [8].

Clinical signs of GTX intoxication reported in both humans and animals include vomiting, frothy salivation, ataxia, limb paralysis, inability to stand, tachypnea, bradycardia, hypotension, tremors, and generalized paralysis [8]. The clinical manifestations observed in the present case were consistent with these previously documented clinical signs, with no notable deviations. Circulatory disturbances such as hypotension and bradycardia are commonly reported in GTX poisoning across species; however, electrolyte imbalances are generally not regarded as characteristic features of this intoxication [13]. In the current case, a mild hypokalemia was observed, whereas sodium and chloride levels remained within reference intervals. Conversely, the recorded hyperglycemia and hypophosphatemia could not be conclusively linked to GTX exposure.

In the present study, identification of the toxic compounds was not performed. Previous investigations in naturally occurring poisoning cases in miniature pigs have detected GTX I in body fluids [14], while experimental studies in goats have identified GTX I and GTX III in feces [4]. Notably, the concentrations reported in these studies were relatively low compared with other reports, suggesting rapid metabolism and excretion of GTXs in vivo [11]. It has been documented that ingestion of fresh leaves amounting to approximately 0.1% of body weight induces toxicity in goats [5], and experimental data from guinea pigs have demonstrated a dose-dependent lethality of GTXs [12]. Experimental studies in guinea pigs have demonstrated that GTX III can induce centrally mediated pulmonary hemorrhage through activation of the sympathetic nervous system and subsequent hemodynamic changes [10]. The marked pulmonary congestion and edema observed in the present case may suggest the involvement of a similar mechanism.

Previous reports of Japanese pieris toxicosis in animals include cases resulting from the accidental administration of manually harvested leaves [2,3,4], as well as incidents of voluntary ingestion by goats [5,6] and miniature pigs [14]. Typically, animals tend to avoid consuming plants of the Ericaceae family, including Japanese pieris, unless subjected to nutritional stress or forage scarcity. Therefore, Japanese pieris poisoning is generally considered to occur primarily under husbandry conditions that differ significantly from the animals’ natural environment or when plant material is deliberately offered as feed [3]. In the present case, the affected animal had recently been introduced to a newly established enclosure. Although alternative palatable forage was available and no leaves were intentionally offered, the characteristic clinical signs, their progression, and the pathological findings strongly indicate that ingestion of Japanese pieris was the causative factor. Given that accidental poisoning may occur abruptly under unforeseen circumstances and can result in severe consequences, strict monitoring and control of toxic plants, including Japanese pieris, are imperative. Moreover, this case underscores the importance of thoroughly assessing zoological exhibits for potential environmental hazards, particularly toxic plants, prior to the introduction of susceptible species. Such preventive measures are crucial to ensure animal health and welfare in captive environments.

## 4. Conclusions

Our report describes possible fatal poisoning owing to ingestion of Japanese pieris in an alpaca. The characteristic clinical signs, along with the gross and histological findings, were consistent with Japanese pieris poisoning. This is the first case report of Japanese pieris poisoning in an alpaca.

## Figures and Tables

**Table 1:** Result of the hematological and biochemical examinations in an alpaca.

	Results	Reference Ranges [7]
Hematocrit	23	17.5–47.6%
Platelets	91,000	0–387,000/mm^3^
CK *	15.0	0.6–14.7 μkat/L
Glucose	362	45–75 mg/dL
Ca	1.9	2.0–2.6 mmol/L
Sodium	146	138–158 mmol/L
Potassium	3	3.95–6.07 mmol/L
Chloride	114	105.0–126.5 mmol/L
Phosphorus	0.26	1.18–3.44 mmol/L

* CK = creatine kinase.

## Data Availability

The data that support the findings of this study are available from the corresponding author upon reasonable request.

## Abbreviations

The following abbreviations are used in this manuscript:

GTXs	Grayanotoxins
HE	Hematoxylin-eosin
